# Editorial Correspondence

**Published:** 1866-03

**Authors:** 


### EDITORIAL CORRESPONDENCE.

The political atmosphere by which one is surrounded at the Capitol is not favorable for the composition of a communication relating only to medical matters, and of interest to the medical profession. We have endeavored, however, to collect some facts at the headquarters of the medical department of the army, and from the last Report of the Surgeon General, which will, we trust, be of interest to our readers.

In no other department of the army has retrenchment been more rapid and judicious than in this. On January 1st, 1865, there were 201 general hospitals open, with a capacity of 136,000 beds. There now remain but 9 general hospitals, with only about 1000 patients in them, and 247 post hospitals, containing 3300 patients. The large amount of medical and hospital supplies which the reduction of the army has rendered no longer necessary, have been or are being sold, and in some cases the articles sell for more than their cost price to the Government. All of the river hospital boats have been turned over to the Quartermaster's Department. Of the 547 surgeons and assistant surgeons of volunteers, appointed since April, 1861, but 23 of the former and 19 of the latter are still in the service; and of the 5523 acting assistant-surgeons, only 246 are now in service. 264 hospital chaplains were appointed during the war, and of these only about 20 remain in commission.

During the war, 34 officers of the medical staff have been killed or died of wounds received in battle, 24 wounded, and 188 have died of accident or disease contracted in the service. 1 died in a rebel prison, and 6 of yellow fever. A complete record may increase this number. Surely no other profession or class of men have brought greater sacrifices to their country's altar than have the medical profession of this country, as is shown by the records of the medical department of the army and navy.

The number of sick and wounded white troops treated in general hospitals alone, from 1861 to July, 1865, was 1,057,423, and the mortality rate of these was 8 per cent. But we need scarcely enter upon the statistics of any part of the medical department, for we have not space for even a very general reference to them, and the profession must be content to wait until the statistics can be completed, and, by the use of all available means, be made reliable.

The materials for the medical and surgical history of the late rebellion are, fortunately, commensurate with the magnitude of the struggle itself; and the zeal, energy and capacity with which the crude matter is being worked up, justifies the prediction, that it will prove as great a success as was the war for the salvation of the Union. No medical man who visits the divisions of the Surgeon General's office, in which this vast work is going on, can, with good reason, offer objections to the measures adopted by the Surgeon General to prevent intrenchments upon that which belongs to the Government, but which will be given to the profession so soon as it can be prepared with that accuracy and minuteness that is necessary to render such a history of value. It is impossible for any man or set of men to prepare a medical and surgical history of the late war so well as can be done under the auspices of the Government, even were the whole records of the medical department thrown open to all who might desire to examine them. The collection of facts, and the perfecting of the history of individual cases, and the preparation of statistics that shall be reliable, requires more labor and expense than any private individual could possibly afford; and it is not at all probable that a work, complete and reliable in all respects, could ever be made from any other source than that from which it is to come. We must be allowed to state, as a farther argument upon this point, that it is not from the records of the medical department alone during the war that data are to be gained, but the records of the Adjutant General's office are consulted in many cases to test the correctness of medical reports; the reports of pension surgeons and of artificial limb makers are consulted, and furnish valuable information as to the results of many of the most interesting cases, and from these reports the address of the men and the pension surgeons are obtained, and when the history of certain cases cannot be completed otherwise, letters of inquiry are addressed to one or the other, or both, and in this manner all the desired information is obtained.

The Army Medical and Surgical Museum is the great object of interest to the medical man here. It furnishes another source of indisputable facts for the medical and surgical history, and is an institution of which the profession of the country should be proud. The surgical specimens now number over 5000, embracing every variety of wounds and injuries, and a history of each case is placed on record. The number of medical specimens is over 800, carefully preserved and ingeniously mounted, so as to be seen to the best possible advantage. A descriptive catalogue of specimens is in course of preparation, and will soon be issued; this will greatly aid visitors, as a concise and satisfactory history of each case from which the specimen was obtained, is given. The general classifications of surgical specimens are 25, with several subdivisions of each. The Museum is open to all, and has become an object of interest not only to the medical profession but to the public generally.

Besides the means already referred to as available for a medical history of the war, we should not fail to mention the interest added to the work by the microscope. Under the supervision of Brvt. Maj. J. J. Woodward, Asst. Surg. U. S. A., micro-photography has been brought to such a state of perfection as to be available for the illustration of micro-pathological anatomy. The microscopical series in the Army Medical Museum is already the largest and best micro-pathological collection in the United States.

In concluding this brief account of the materials and of the labor that is being done for the medical and surgical history of the war, we would admonish our readers to be patient, for at no distant day, if Congress does its duty in making appropriations, a work will be placed in the hands of the profession of which every worthy member may well be proud. Never before has such a mass of materials been furnished for such a history, and time must be allowed to digest them; and yet we venture the prediction, that there will not be nearly the time consumed in their preparation that has been consumed in producing all similar works in other countries.

The work is in good hands; it is open yet for the contributions of professional men and medical officers of the army, and due credit will be given to any one who, in any manner, aids in the great enterprise. R. M. L.

WASHINGTON, D. C., Feb. 28th, 1866.

